# Isolation and Bacteriocin-Related Typing of *Streptococcus dentisani*

**DOI:** 10.3389/fcimb.2019.00110

**Published:** 2019-04-16

**Authors:** Georg Conrads, Jacqueline Westenberger, Martha Lürkens, Mohamed M. H. Abdelbary

**Affiliations:** Division of Oral Microbiology and Immunology, Department of Operative and Preventive Dentistry and Periodontology, RWTH Aachen University Hospital, Aachen, Germany

**Keywords:** *Streptococcus oralis* subspecies *dentisani*, probiotic, dental caries, bacteriocin (s), isolation procedure, subtyping

## Abstract

*Streptococcus oralis* subspecies *dentisani* is explored as an anti-cariogenic probiotic. Here, subjecting freshly stimulated saliva samples of 35 healthy volunteers, six epidemiologically unrelated and two related strains were isolated (prevalence around 20%) applying a newly developed three-step procedure. Furthermore, the probiotic strain *S. dentisani* 7746 (AB-Dentisanium®) was tested under a variety of environmental conditions for its inhibitory effect on six *S. mutans*, two *S. sobrinus*, 15 other oral or intestinal streptococci, 15 *S. dentisani* strains, and six representatives of other species including periodontopathogens. All except one of the *S. mutans* strains were inhibited by 7746 colonies or culture supernatant concentrate but only if either the test cell number was low or the producer or its bacteriocin concentration, respectively, was high. *S. sanguinis* OMI 332, *S. salivarius* OMI 315, *S. parasanguinis* OMI 335, *S. vestibularis* OMI 238, and the intestinal *S. dysgalactiae* OMI 339 were not inhibited, while the other 10 streptococcal strains (especially *S. oralis* OMI 334 and intestinal *S. gallolyticus* OMI 326) showed a certain degree of inhibition. From the panel of other bacterial species only *Aggregatibacter actinomycetemcomitans* was slightly inhibited. With the exception of OMI 285 and OMI 291 that possessed a 7746 bacteriocin-like gene cluster, all *S. dentisani* strains and especially type strain 7747^T^ were strongly inhibited by 7746. In conclusion, probiotic strain 7746 might antagonize the initiation and progression of dental caries by reducing *S. mutans* if not too abundant. *S. dentisani* strains inhibit each other, but strains with similar bacteriocin-related gene clusters, including immunity genes, are able to co-exist due to cross-resistance. In addition, development of resistance and adaptation to 7746-bacteriocins was observed during our study and needs attention. Hence, mechanisms underlying such processes need to be further investigated using omics-approaches. On the manufacturing level, probiotic strains should be continuously tested for function. Further clinical studies investigating inhibition of *S. mutans* by AB-Dentisanium® are required that should also monitor the impact on the oral microbiome composition including resident *S. dentisani* strains.

## Introduction

Due to the dramatic increase of antimicrobial resistance, many efforts are made to reduce the prescription of antibiotics and to change from therapy to prevention. Probiotics might help to prevent infectious diseases or microbial dysbiosis, with dental caries as a popular target because of its high prevalence and risk of sequelae (Global Burden of Disease Study, 2015). For example, *Streptococcus salivarius* subspecies *salivarius* strains K12 and M18 (Mia) were explored as anti-pharyngitis, anti-caries and anti-halitosis probiotics (Horz et al., 2007; Wescombe et al., 2012; Di Pierro et al., 2015). *S. mutans* JH1140, as another example, was made lactate dehydrogenase deficient by deletion of an alcohol dehydrogenase gene, while a gene of *Zymomonas mobilis* was introduced to re-constitute the metabolic fitness. This double-mutated strain was found to still colonize the teeth but was significantly less cariogenic in rodent models of dental caries (Hillman et al., 2000). A clinical phase-1b-study subjecting this double mutant in the SMaRT replacement therapy was started but discontinued due to candidate enrollment difficulties (www.oragenics.com/ technology-pipeline/lbp/smart, last access 25.01.2019). Natural lactate dehydrogenase deficient mutans-streptococci or super bacteriocin producers were shown to replace cariogenic strains (Hillman et al., 1987, 2009). Jindal et al. recorded a statistically significant reduction (*p* < 0.001) of salivary mutans streptococci counts in a test group taking Sporolac®-capsules, containing cells of sporulating, lactic acid producing *Bacillus coagulans* for 14 days (Jindal et al., 2011).

Combinations of probiotic strains were also studied and already marketed. Jindal et al. exhibited a reduction of mutans streptococci in a test group taking Darolac®-capsules containing *Lactobacillus rhamnosus, L. acidophilus, Bifidobacterium longum*, and the yeast *Saccharomyces boulardii* for 14 days (Jindal et al., 2011). Furthermore, a mouthwash (ProBiora^TM^) is on the market that contains three specific strains of naturally occurring oral bacteria, namely *S. oralis* KJ3, *Streptococcus uberis* KJ2, and the spontaneous lactic acid-deficient variant of *Streptococcus ratti*, strain JH145. When administered twice daily over a period of 4 weeks, a substantial decrease in the levels of dysbiotic bacteria (*S. mutans* and several periodontal pathogens) were observed while safety issues were not noted in a small clinical trial (Zahradnik et al., 2009).

The central problem regarding anti-caries probiotic activity is, however, that origin and site of intended action is highly different. All the above mentioned anti-cariogenic strains, except *S. oralis* KJ3, are either genetically engineered or not colonizers of human teeth. In the case of *S. salivarius* its primary habitat is saliva (and the gut) but not the dental biofilm and it does not persist on tooth surfaces (Horz et al., 2007). Many probiotic strains are fecal isolates and some are even of animal (feces or milk) origin. From the “Microbes for Probiotics” list of the ATCC collection containing 24 strains of genera *Bifidobacterium, Enterococcus, Lactobacillus*, and *Lactococcus*, only *Lactobacillus casei* ATCC® 15008 is specified as isolated from the human mouth.

A second problem for a very limited or even failed anti-caries activity is the potential demineralization effect of oral probiotics by lowering the pH when attaching to teeth. For instance, when *Lactobacillus salivarius* W24 was tested in an oral biofilm model, it further lowered the pH and affected the compositional stability of the oral community (Pham et al., 2009). Some of the lactic acid producing probiotic strains might even be cariogenic (Badet and Thebaud, 2008; Mantzourani et al., 2009). In summary, there is insufficient evidence supporting the use of established probiotics to manage, prevent or treat caries (Gruner et al., 2016).

As a solution to the above mentioned problems, López-López introduced *Streptococcus oralis* subspecies *dentisani*—abbreviated to *S. dentisani*—strain 7746 as a probiotic with a triple anti-caries action as (i) it is a colonizer of human teeth, (ii) it buffers in the presence of arginine acidic pH through an arginolytic pathway, abbreviated to ADS (Velsko et al., 2018), and (iii) it inhibits the growth of major oral pathogens through the production of potent bacteriocins (López-López et al., 2017). The authors proposed the use of *S. dentisani* 7746 as a promising probiotic against tooth decay and brought it to market (patent WO2012028759, AB-Dentisanium®, Ab-Biotics, www.ab-biotics.com/products-en/probiotics-en/oral-care-en/ab-dentisanium-en.html, last visit 21.01.2019). However, while the buffering activity of *S. dentisani* was confirmed (Conrads et al., 2018), so far no independent study evaluated the reduction of mutans streptococci counts (neither *in viv*o nor *in vitro*) or investigated the impact of probiotic *S. dentisani* on other bacteria including naturally occurring *S. dentisani* strains. In the worst case, strain 7746 could out-compete a resident anti-cariogenic *S. dentisani*-like strain and then disappear due to failed adaptation to the host. It could also interfere with streptococcal strains and species that naturally keep homeostasis and antagonize *S. mutans* (Huang et al., 2018; Thurnheer and Belibasakis, 2018).

To study these questions we first isolated eight *S. dentisani* strains with a newly developed protocol and tested the bacteriocin-related antimicrobial activity of strain 7746 against (i) a collection of *S. mutans*-strains (the principal target of the probiotic), (ii) two *S. sobrinus*-strains (another caries-associated mutans-streptococcal species), other oral and intestinal streptococci, (iii) *S. dentisani* itself (15 test strains subjected), and (iv) other non-streptococcal oral bacteria, including periodontitis-associated species. Furthermore, we describe different bacteriocin-related gene-clusters and a derived multiplex PCR to genetically type *S. dentisani* isolates. The cluster-type is related to bacteriocin-production and immunity. Both are important factors determining co-existence of strains.

## Materials and Methods

### Isolation of *S. dentisani* From Saliva of Human Volunteers

**Figure 1:**
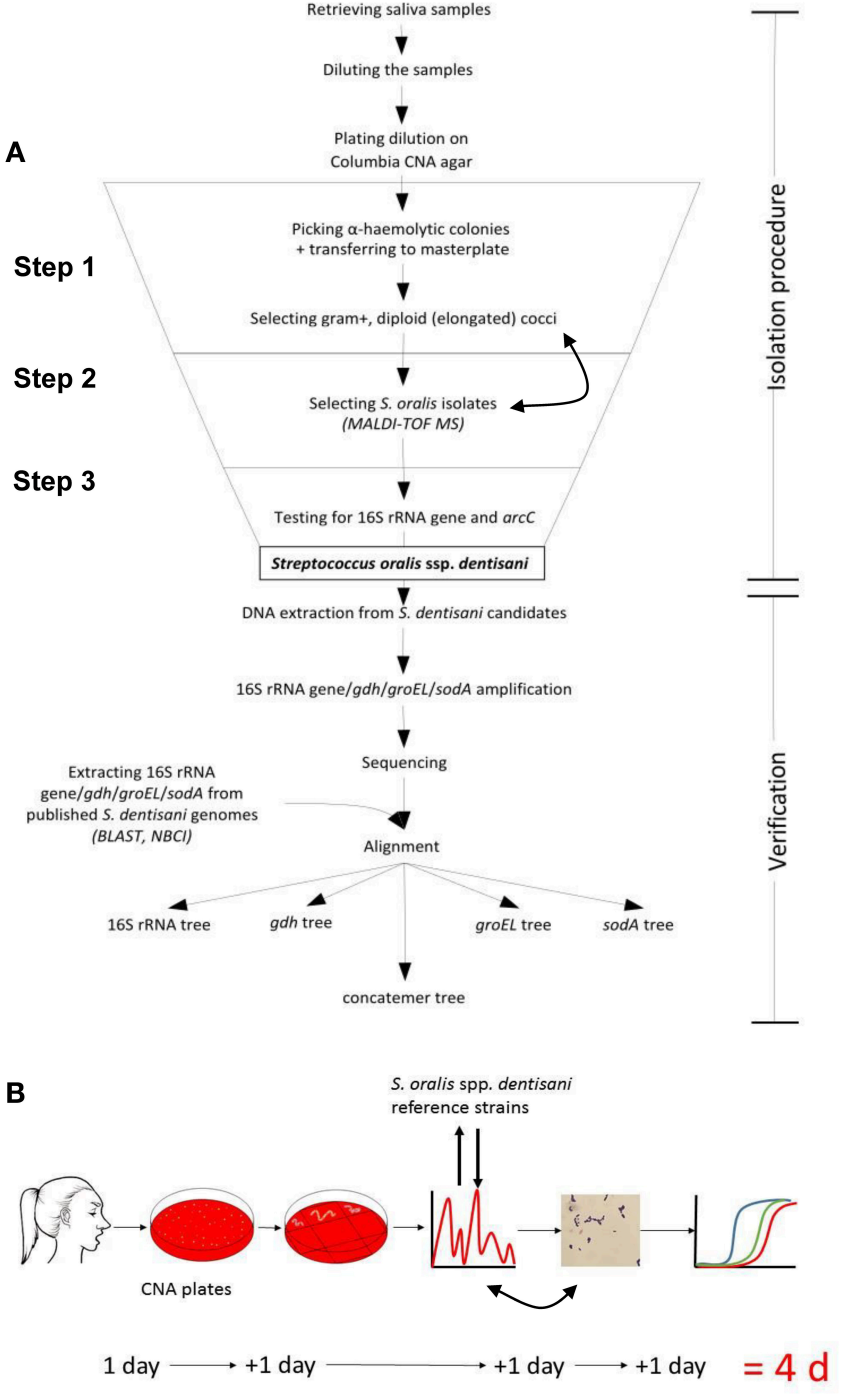
**(A)** Flowchart and **(B)** time-line for the isolation and verification procedure for human *S. dentisani* strains. CNA: Columbia colistin-nalidixic-acid blood agar plate (selective for gram-positive cells). Double arrows indicate that the MALDI-TOF and gram-staining sequence is interchangeable.

A number of 35 caries-free and healthy volunteers were informed about the study and signed an informed consent that had been approved in accordance with the guidelines of the Ethics Committee of the University Hospital Aachen, Germany and the Declaration of Helsinki. Of each donator 1 mL freshly produced saliva was collected. Salivary flow was stimulated by chewing paraffin pellets. The isolation and verification of *S. dentisani* candidates from these samples was performed in three steps (see Figure 1 for overview): (1) selection of gram-positive α-hemolytic bacteria with a colony and cell-morphology matching with the reference strains 7746 and 7747^T^, (2) applying matrix assisted laser desorption ionization-time of flight mass spectrometry (MALDI-TOF MS) for selecting “*S. oralis*” isolates with a similar profile of ribosomal proteins, (3) functional screening for carbamate kinase gene (*arcC*) and a *S. dentisani* species-specific 16S rRNA gene variant (V1-V3 region).

Description of the 3-step procedure in detail (Figure 1):

Step 1: Saliva was homogenized by vortexing and 2.5 μL were mixed with 247.5 μL 0.9% NaCl solution. Next, 2.5 μL of the respective dilution was transferred into 497.5 μL 0.9% NaCl followed by plating the whole 500 μl preparation on a selective Columbia CNA blood agar (Becton Dickson, containing 10 μg colistin and 5.5 μg nalidixic acid per ml and 5% sheep blood) and incubation overnight at 37°C in 7–8% CO_2_ air atmosphere. Alternatively, by using colistin discs, non-selective blood agar plates can be used for isolation from the inhibition zone but this needs a second passage to ensure clonality. After 24 h, single α-hemolytic colonies were picked and transferred onto a Trypticase-soy-blood agar (TSBA) master plate. Reference colonies (7747^T^ and 7746) appeared glossy with a white to a dark gray shimmer after 12 h with the circular and convex shape of a little water drop or, after 24–48 h, umbonate (button-like) (Supplementary Figure 1). Such respective colonies were predominantly picked. Colonies with a different morphology (e.g., dry or even crumbly) were also tested to make sure not missing any colony variant, but were later not confirmed as *S. dentisani*. The standard gram staining method was used (before or even after MALDI-TOF, Step 2, as staining is more labor intense) to determine the cell morphology of each isolated *S. dentisani* candidate. Candidates were mostly diploid but were also found arranged in short chains, elongated to bean-shaped, gram-positive to gram-variable, partly plump cocci or coccoid rods. The cell morphology differed slightly between strains but e.g., long chains or round, non-elongated cocci were not found among the candidates (Supplementary Figure 1).
Step 2: MALDI-TOF MS (Biotyper, Bruker Daltonik) was used to further specify the isolates. Single colonies of the prepared master plate were picked using a sterile toothpick, inoculated onto the MSP 96 target next to a BTS standard and mixed with the HCCA matrix dissolved in 250 μL Bruker standard solvent. *S. dentisani* strains 7746 and 7747^T^ served as reference. The FlexControl 3.4 program was used for the evaluation of spectra. As the current Bruker database (status 12-2018) does not list *S. oralis* subspecies *dentisani*, all “*S. oralis*” entries together with 7746 and 7747^T^ protein profiles were used as reference. Colonies to which mainly *S. oralis* (excluding e.g., *S. mitis*) references were assigned were considered as *S. dentisani* candidates. A high number of hits as “*S. oralis”* was crucial for correct specification. To ease identification, the inclusion of *S. dentisani* reference strains into the Bruker database is desirable.
Step 3: “*S. oralis*” isolates were further analyzed by performing quantitative real time PCR with two primer sets: one specific for the 16S rRNA gene variant (SDent-16S-F: 5′TGAAGGAGGAGCTTGCTTCTC3′, SDent-16S-R: 5′CAAACAGTTATCATGCAATAACTG3′, lenght 137bp, deduced from the V1-V3 region) of *S. dentisani* and the other specific for the carbamate kinase gene *arcC* (CkSdAlt-F: 5′ AATGTGCTCTTGATAACGCATTRC 3′, CkSdAlt-R: 5′ GGTTGCTCCTGTTTTTTCGCTC 3′), the latter component of the ADS. The primers as well as PCR conditions were described earlier by our group (Conrads et al., 2018). Isolates that were tested clearly positive for both genes were effectively classified as *S. dentisani*. For verification, the 16S rRNA-gene (V1-V3, *E.coli* position 27-557) and three other housekeeping genes that are known for their species-specific resolution of streptococci, namely *gdh* (coding for glucose-6-phosphate 1-dehydrogenase, gene position 745 to 1404), *groEL* (coding for a heat shock protein, molecular chaperone GroEL, gene position 555 to 1312), and *sodA* (coding for a superoxide dismutase, gene position 36 to 471) were amplified and sequenced (Conrads et al., 2017). A phylogenetic tree based on these concatemeric genes (order 16S rRNA, *gdh, groEL*, and *sodA*) is able to separate *S. oralis* subspecies, leaving *S. mitis* and *S. infantis/S. peroris* as distinct clusters. Such a tree, including all *S. dentisani* isolates and reference strains subjected in this study, is provided as Supplementary Figure 2.

### Testing Bacteriocin Activity

Inhibition experiments were carried out with cultures, but also with concentrated supernatants of *S. dentisani* 7746 and 7747^T^ and clinical isolates. Different methods and conditions were applied.

#### Deferred Antagonism Test

The deferred antagonism test was originally described by Tagg and co-workers (Tagg and Bannister, 1979; Hyink et al., 2007). In our modified deferred antagonism method, four colonies of the producer strains (subjecting mainly *S. dentisani* 7746 but also 7747^T^, OMI 105, 116, 166, 168, 214, 215, 284, 285, 287, 290ab, and 291) were picked and inoculated by the means of a 10 μl-loop vertically forming two 0.5 cm wide streaks with a distance of 2.0 cm on a non-selective Columbia blood-agar (CBA) plate. The plate was incubated for 24 h at 37°C and 7–8% CO_2_. After incubation, the producer biomass was removed by the aid of a thin glass cover slide and/or a sterile swab and the few remaining cells sterilized with chloroform vapors for 30 min in a sealed plastic bag. The vapor source was 10 mL chloroform deposed onto a dental cotton role. Evaporation of the remaining chloroform until 15 min was ensured before the test strains were applied in the following workflow. A few (1–4 depending on the size) colonies of *S. mutans, S. sobrinus, S. dentisani*, other streptococci or other bacteria (see Supplementary Table 1: test strains) were first suspended in 100 μL 0.9% NaCl and 2.5 μL of the suspension was released in the middle between the two 0.5 cm wide producer zones, and streaked from the middle first to the right and then to the left producing decreasing colony densities. This scheme is illustrated in Supplementary Figure 3A. After incubation (24 h at 37°C and 7–8% CO_2_), the inhibitory effect of the released bacteriocins in the producer zone became apparent by abrupt gaps in bacterial growth or inhibition zones, respectively, which is illustrated in Supplementary Figure 3B.

#### Inhibition Assays (Solid and Liquid) With S. Dentisani Supernatants

Concentrated supernatants of *S. dentisani* 7746 and a few others (7747^T^, OMI 284 and 285; further on named “producer”) were obtained according to the protocol of López-López et al. (2017). Briefly, a single colony of a fresh overnight passage of the strain was inoculated into 50 mL of brain-heart-infusion (BHI) broth and incubated aerobically at 37°C and 7–8% CO_2_ without agitation until an optical density round about 1.5 (OD 610 nm) was reached. This broth culture was twice-centrifuged at 4,000 rpm for 10 min and the cell-pellets discarded. The supernatant was filtered through a Millex®GP filter with a pore size of 0.22 μm to remove any remaining cell or clumps. The supernatant was 10-fold concentrated at either 30, 45, and 60°C in the Concentrator 5301 (Eppendorf). Different temperatures were used to examine the heat stability of the bacteriocin and to avoid overheating and denaturation in any case. The concentration time was approximately 2:00 h at 60°C, 3:15 h at 45°C, and 4:00 h at 30°C with variation depending on the number of tubes and the starting volume. All concentrates were directly used for susceptibility testing or stored at −20°C until use, the latter reducing the activity only slightly over time.

For testing on solid medium, single to very few colonies of all test strains (Supplementary Table 1) were suspended in 500 μL 0.9% NaCl and poured on different agar plates (CBA, TSBA, Mueller-Hinton [MH] agar and BHI-agar, kept at room temperature overnight and dry to ease complete absorption of suspension). The suspension was uniformly distributed over the agar surface by slewing—only if necessary—by aid of a Drigalski-spatula. After full-fledged drying, a volume of 5 μL of the producer supernatant concentrate was carefully pipetted onto the inoculated agar plate and incubated at 37°C and 7–8% CO_2_ for 18–24 h until confluent colonies (and inhibition) were observed. To investigate the contribution of antimicrobial peptides and H_2_O_2_ to the overall inhibition, 1U of proteinase K (Merck) or 1U of horse-radish peroxidase (Sigma-Aldrich) was added to the concentrate and incubated at 37°C for 5 min.

For testing the inhibition of selected strains (7747^T^, six *S. mutans* strains and two *S. sobrinus* strains, Supplementary Table 1) in broth medium with and without horse blood as additive, a mixture of (i) 980 μl BHI or (ii) 915 μl BHI plus 65 μl horse blood, and 20 μl of a bacterial suspension was prepared. The suspension was made from a fresh overnight culture adjusted to 0.5 McFarland and 1:50 diluted. In 96-well-plates, 50 μl of the mixture (inoculum) was prepared with 50 μl 10-fold concentrated producer supernatant-concentrate to get the first (1:2) dilution. For further dilutions the concentrate was serially reduced and replaced by BHI until a final concentration of 1:512 was reached, keeping always the same inoculum. The 96-well plates were covered with sealing-film and incubated at 37°C and 7–8% CO_2_ for 24 h. After incubation, the minimal inhibitory concentration of the producer supernatant was determined and defined as the highest dilution of the supernatant which prevented visible growth of the test strain.

### Typing *S. dentisani* Strains Based on Bacteriocin-Related Genes

To further evaluate the anti-caries repertoire (bacteriocin production, transport of bacteriocins and immunity, arginine metabolism for re-alkalization) encoded in known *S. dentisani* genomes a comparison of the bacteriocin clusters was conducted using the BAGEL4 online tool (van Heel et al., 2018). Members of *S. oralis* genomo-subspecies 1 (ATCC 6249-like) were added as outgroup. These strains seem to be phylogenetically very closely related but not identical to *S. dentisani* (Jensen et al., 2016). Additionally, we analyzed 17 publicly available *S. oralis* subspecies *dentisani* and *S. oralis* genomo-subspecies 1 genomes that were obtained from the National Center for Biotechnology Information (NCBI) database. For background, BAGEL4 enables researchers to mine bacterial (meta-) genomic DNA for bacteriocins and other ribosomally synthesized and post-translationally modified short peptides (RiPPs) and related genes. It takes advantage of the fact that bacteriocin genes are commonly located in proximity of genes encoding proteins needed for processing, modification, transport, regulation and/or immunity. An independent ORF detection algorithm prevents the oversight of small, non-conserved ORFs, which are the most probable candidates for bacteriocin genes. The knowledge based search allows a much faster identification of putative bacteriocin-producing strains than the classical way, the laborious testing of the putative producer strains (de Jong et al., 2006). The database, containing almost 500 RiPPs, 230 unmodified bacteriocins and 90 large bacteriocins, enables the prediction of promoter and terminator sites also (van Heel et al., 2018). The comparison of the obtained clusters was performed via Mauve 2.4.0 (Darling et al., 2004) and BRIG 0.95 software (Alikhan et al., 2011) using the cluster extracted from the 7746 genome as reference. Prior to the multiple alignments, each detected cluster was individually reordered against the 7746 cluster using the MoveContigs function of Mauve. The reordered clusters were then aligned using progressiveMauve (Darling et al., 2010). Based on the comparative genomics analysis of different bacteriocin-clusters (some containing ADS genes as well) (see **Supplementary Data File “BAGEL typing”)**, marker genes were chosen for developing a multiplex PCR for the subtyping of *S. dentisani*. Using Primer3Plus (Untergasser et al., 2007), eight different primer sets were designed targeting the selected genes with a product size range between 153 and 1,089 bp. The multiplex PCR, applying two different primer mixtures (Table 1), was executed as follows: initial denaturation for 10 min at 94°C; 30 cycles: denaturation at 94°C for 30 s, annealing at 52°C for 30 s, elongation at 72°C for 60 s; final elongation for 10 min. With these two multiplex PCRs a typing of all *S. dentisani* isolates was possible and meaningful results of bacteriocin clusters were achieved.

**Table 1:** Primer pairs designed for the mutliplex PCR for bacteriocin cluster typing of *S. dentisani*.

**Primer**	**Target**	**Sequence**	**Amplicon size**	**Ta [°C]**	**Primer Mix**
S.d-Blp_F S.d-Blp_R	Bacteriocin BlpN/K of class IIb	CTTGCGTTGAAGGTGGAGAT GCTCCCCAAGCTGCTGTA	160	57	1
S.d-Pnc_F S.d-Pnc_R	Bacteriocin PncT of class IIb	GACAATTAGTGGTGGAGATGACTG GGAACTGGAGGTGCAGGAT	153	56	2
S.d-Arg.syn_F S.d-Arg.syn_R	Argininosuccinat synthase	CGCTGTTTGTATGGATGTGG ATTTCCGAGCCACTACCTGA	968	56	1
S.d-Arg.lyase F S.d-Arg.lyase R	Argininosuccinat lyase	AAGAGGCAGAGCAGATCCAA TCAAGGGATGGTAGGTTTGC	1,089	56	2
S.d-Lan-effpr F S.d-Lan-effpr R	Lantibiotic efflux protein	ACGCTCTGACGTTTCTGACA CTCCCATTGACTCCTCAGGT	534	56	1
S.d-Mu-lik-pr F S.d-Mu-lik-pr R	Transcriptional regulator	CTTCGACGAGTTTGGACACA ACATCGTATGGACGGAGCAT	510	56	2
Lac1- F Lac1 R	Lactococcin1	AAATTGGTGTCGCAACCGTA TCTAATCCTGTTGGCGGAAT	250	58	1
Lac2- F Lac2 R	Lactococcin1	GTGGTYTGCTTGCTACGACA TCCTGTTGGCGGAATTTTAG	218	56	2

## Results

### Isolation of *S. dentisani* From Saliva of Human Volunteers

We detected eight new *S. dentisani* strains among 250 colistin- and nalidixic acid resistant α-haemolytic streptococci that were retrieved from saliva of 35 volunteers. Among these, two strains (OMI 290a and 290b) were isolated from the same volunteer. The comparison of 16S rRNA (V1-V3), *gdh, groEL*, and *sodA* sequences revealed that both strains are genetically highly similar even though not identical (Supplementary Figure 2). In addition, two other *S. dentisani* isolates (OMI 284 and OMI 285) were isolated from another volunteer but clustered distinctly in the concatemeric tree based on quite a few nucleotides, especially in the *groEL* housekeeping gene (Supplementary Figure 2). Taken together we isolated seven genetically distinct and six epidemiologically unrelated *S. dentisani* strains from six out of 35 volunteers with a prevalence of around 20% in this caries-free cohort. All new *S. dentisani* along with the two reference strains, four *S. dentisani*-endocarditis isolates (OMI 105 [SN 54788], OMI 116 [SN 39325], OMI 166 [SN 58364], OMI 168 [SN 54787]), and two dental plaque isolates (OMI 214 and OMI 215) from a previous study (Conrads et al., 2018) are listed in Supplementary Table 1 and their colony- and cell-morphology is represented in Supplementary Figure 1.

### Testing Bacteriocin Activity of *S. dentisani* 7746 to Inhibit *S. mutans*

Through the deferred antagonism test, an inhibition of all *S. mutans* strains except R658 was observed but only in areas of low test strain colony density. The inhibition could be improved by choosing a suboptimal growth temperature (30°C) for the test strain (illustrated in Supplementary Figure 3B). However, under optimal growth conditions and for high colony density of *S. mutans*, no inhibition was detected and discovered independent of the strain.

Testing the effect of concentrated 7746-supernatant on solid agar media, inhibition could only be demonstrated for *S. mutans* UA159 after further weakening the cells by addition of Tween 80. In the broth assay with serially diluted 10-fold 7746 culture-supernatant (1:2 down to 1:512) without 3% horse blood, the *S. mutans* strains ATCC 25175^T^, UA159, and AC 4446 were inhibited by the 1:2 dilution and strains KK21, KK23, and R658 by the 1:4 dilution. In the same assay but with 3% horse blood, most *S. mutans* strains were inhibited until the 1:4 dilution, except strain KK21 which was more susceptible (1:8) and strain R658 which was more resistant (1:2). *S. sobrinus* ATCC 33478 was inhibited by the 7746 culture-supernatant at the 1:4 dilution and AC 163 at the 1:2 dilution for both assays, with and without horse blood. As originally described this activity was completely destroyed by proteinase K but not by peroxidase-treatment, proving the proteinous nature of the inhibitor and excluding H_2_O_2_ as agent. The undiluted 10-fold BHI concentrate (control) inhibited all test strains by its sheer ionic strength in contrast to the non-inhibitory 1:2 dilution.

### Testing Bacteriocin Activity of *S. dentisani* 7746 to Inhibit Other *S. dentisani* Strains

In contrast to the weak inhibition of *S. mutans, S. dentisani* 7747^T^ was inhibited by *S. dentisani* 7746 in the deferred antagonism test and by the 7746 culture-supernatant on all solid media and in broth media even at 1:32 (with) or at 1:64 (without 3% horse blood). Testing 13 genetically confirmed *S. dentisani* strains (Supplementary Table 1 including the eight new isolates), only the strains OMI 285 and OMI 291 were resistant to the 7746-bacteriocin-bearing-supernatant. All other *S. dentisani* strains were found to be susceptible to various degrees.

Using 7746/7747^T^ as bacteriocin activity model, we performed additional tests to investigate (i) the best conditions for producing and maintaining 7746-bacteriocins and (ii) the development of resistance or adaptation against 7746-bacteriocins. Strain 7746 produced bacteriocins most efficiently at pH 6.97 but not at 6.16 or above 7.94 (Supplementary Figure 3C). The 7746-supernatant was concentrated under three different temperatures. The best concentrate of active bacteriocins was obtained between 30 and 45°C. Higher temperatures such as 60°C sped up the concentration process but with minor loss of bacteriocin activity (Supplementary Figure 3D). We also tested different agar media and techniques for revealing bacteriocin activity (Supplementary Figures 3E,F). The diffusion of bacteriocins was slightly reduced in blood free medium such as MH, leading to smaller inhibition zones. This was observed when the supernatant was applied on discs or in stamped troughs while the inhibition zone was larger when the bacteriocin-preparation was applied directly onto the agar surface. Regarding development of resistance or adaptation, after a few sub-cultivations, an increasing number of unsusceptible 7747^T^-colonies were observed. We isolated in total eight different 7747^T^-progenies (see Supplementary Table 1) that showed either single resistant colonies within a pronounced inhibition zone (OMI 278, Supplementary Figure 3G) or partial (OMI 282, Supplementary Figure 3H) or complete (OMI 276-77, OMI 279-81, OMI 283) resistance with a small or no inhibition zone. By applying ORF540-directed PCR (7746 positive, 7747 negative according to Conrads et al., 2018) we could exclude that the resistant or adapted colonies were 7746-contaminants from insufficient filtration.

### Testing Bacteriocin Activity of *S. dentisani* 7746 to Inhibit Other Species

We tested 12 other oral and three intestinal streptococcal species for susceptibility to the 7746 supernatant (Supplementary Table 1). Five strains were not inhibited at all (oral *S. sanguinis* OMI 332, *S. salivarius* OMI 315, *S. parasanguinis* OMI 335, *S. vestibularis* OMI 238 and the intestinal *S. dysgalactiae* OMI 339), while the majority of 10 strains showed some inhibition with the largest zones for oral *S. oralis* OMI 334 and intestinal *S. gallolyticus* OMI 326. Taken together these findings demonstrate that the probiotic strain 7746 has potential to influence the natural constitution of the oral and intestinal streptococcal community.

From the six non-streptococcal species tested, only *Aggregatibacter actinomycetemcomitans*, a periodontal pathogen, was very weakly inhibited while the other, including *Prevotella intermedia* and *Fusobacterium nucleatum*, showed no inhibition, contradictory compared to earlier results (López-López et al., 2017). However, as the bacteriocin susceptibility might be more strain/clone than species dependent, varying results can be expected.

### Testing Other *S. dentisani* Isolates for Bacteriocin Production

Finally, we tested the bacteriocin activity of other *S. dentisani*-isolates in deferred antagonism tests (7747^T^, OMI 105, 116, 166, 168, 214, 215, 284, 285, 287, 290ab, and 291) and/or with culture concentrate (OMI 284 and 285). The isolates OMI 285 and OMI 291 inhibited 7747^T^ comparable to the probiotic strain but not 7746 itself proving—combined with results from above—cross-resistance between OMI 285, 291, and 7746. Strains OMI 284 and OMI 285, isolated from the same proband, showed also cross-resistance. *Streptococcus dentisani* OMI 287 inhibited *S. mutans* UA159 and *S. dentisani* OMI 168 inhibited *S. mutans* AC446. Finally *S. dentisani* OMI 116 inhibited 7747^T^.

### Typing of *S. dentisani* Isolates by Multiplex PCR of Bacteriocin Cluster-Genes

Derived from bacteriocin cluster analysis by BAGEL4 (based on 17 publicly available *S. oralis* subspecies *dentisani* and *S. oralis* genomo-subspecies 1 genomes, **Supplementary Data File “BAGEL typing”**), multiplex PCRs were designed for typing isolates applying two different primer mixtures. Supplementary Figure 4 represents the typing-results and the presence/absence of certain bacteriocin- but also ADS-related genes. Interestingly, the results revealed that *S. dentisani* strains possess very different combinations of bacteriocin- or bacteriocin-related genes. Despite the variability, exactly the same pattern was found in strains OMI 290a and 290b, not surprisingly as isolated from the same individual and identical by gene sequencing (16S, *gdh, groEL, sodA*). The strains OMI 284 and OMI 285 were also isolated from the same person, but did not demonstrate a close relationship, neither in the phylogenetic tree (Supplementary Figure 2) nor in the bacteriocin-related gene cluster (Supplementary Figure 4). However, as bacteriocin-related operons have a very fluid structure (Miller et al., 2016) the number of bacteriocin-types is sheerly endless and the PCR can provide only some orientation.

The BAGEL4 results regarding the probiotic strain 7746 (see **Supplementary Data File “BAGEL 7746”**) allowed for the identification of bacteriocin-like genes in ORF24, 25, 28, 30, 34, 37, 45, 50, and 59 (encoding nine BlpIIb-like bacteriocins), in ORF57 (encoding PncT), and in ORF42 (encoding a peptide similar to bovicin) (Table 2). The rough BAGEL4-bacteriocin-annotation was supplemented by additional BLAST-searches and by exact Blp/Pnc amino acid sequence information from Miller et al. (2016). The bacteriocin-IIb-genes, short heat-resistant peptides cooperatively acting in pairs, are likely to be responsible for the antimicrobial activity in *S. dentisani*. The three strains (7746, OMI 285 and 291) possessing ORF45 coding a certain BlpNK bacteriocin (Table 2), also shared ORF48 and 53, both coding for a Pnc-related-immunity protein. Locus ORF48 was described before as “ORF540” by López-López et al. (2017) and seems to be indicative for 7746-like (probiotic) strains (Conrads et al., 2018). Interestingly, strains 7746, OMI 285 and OMI 291 did not inhibit each other (see above), while inhibiting all other *S. dentisani*-strains except OMI 284, the latter isolated from the same individual as OMI 285 and thus adapted for co-existence with such strains. Interspersed between the bacteriocin-genes, we found further immunity genes, one encoded by ORF36 and 100% identical with an enterocin A immunity gene (WP_038804501.1), one encoded by ORF43 and 99% identical with an immunity gene of *S. mitis* (WP_125451482.1), as well as three bacteriocin-transporter genes (ORF32 [accessory], 63 [*comA*], 65 [*comB*]). We performed an intense search for regulatory proteins and found *comCDE* about 400,000 bp downstream of the bacteriocin cluster located on the same contig (GenBank CAUJ01000007.1) indicating that the bacteriocin expression of this probiotic strain is regulated by competence.

**Table 2:** Leader and mature putative class II bacteriocin and ComC amino acid sequences of *S. dentisani* 7746.

**ORF within BAGEL4 cluster**	**Orthologs in *S. pneumoniae***	**Leader aa sequence**	**Mature bacteriocin aa sequence RK positive charged, *DE* negative charged aa**	**Net charge protParam^§^**
ORF24	BlpE	MFDYKIVDNQELSNISGG	GLGG***D***VVVGALSGAFQAGQSCIAGGPQAYLICATG GAIVGGILAFGL**R**PP**K**	+1
ORF25	BlpD	MNTKMMEQFKIMDTEMLASIEGG	T***D***WGTVG**K**GAVYGAGIGVAMCTVGGLLTGGSAWAM TAGCAWAGA**K**LGGAFTAIA***D***NIWP	0
ORF28	BlpN	MLSEIYGG	NSGGAAVVAALGCAAGGV**K**YG**K**FLGPWGAAIGGIG GALICGYLAYSATS	+2
ORF30	BlpM	MDTKMIEQFHEMDITMLSSIEGG	**K**NNWQTNVL***E***GGGAAFGGWGLGTAICAASGVGA PFMGACGYIGA**K**FGVALWAGVTGATGGF	+1
ORF34	BlpN, BlpK	MNTKMMEQFESMDTDMLACVEGG	**KK**FG***D***C***E***TAISAGIGVGAVFAGPWGAVGLGTVTNMFFC ATPVS	0
ORF37	BlpJ	MNLKMMEQFEIMDTEMLASKVGG	**K**TIYYGNGLYC***D***NS**K**GCWVNWP***E***AIN**K**ILTNSIVNGFS GGNAGWNSGGPL	+1
ORF42	Hypothetical bacteriocin^*^	MEQSVNNFFILSDEKLTTITAG	AV***E***GISLCMQTIPFPTPQIYLICAAGGAAASVLWPH	−1
ORF45^$^	BlpN, BlpK	MDTDMLACVEGG	***D*****K**VGAG***E***VVQALGVCTIGGAALGSVIPVVGTLAGGILG AQFCTAAWGAL**R**AS	0
ORF50	BlpJ	MNTKMMEQFEIMDTDMLAKVEGG	FGGWG***D***MIAGLLGGLAPSPTL***D***QLNG**K**WPIIHFS**K** PCGPYGIGGTPNSCNG	0
ORF57^$^	pncT	MKKIDYIALNEVELETISGG	***DD***CFIG***D***IGCIGWGIL**K**SIGGMI**K**PGPYVPPVCIP**K**SSW NPAPPVPC	0
ORF59	BlpM, BlpQ	MNTKMMSQFSVIDNEMLDRIEGG	IFGV***DD***AVFWTVGGYVVG**R**IV***D***TAIG***D***FTNQC**RK**NP HQWFCV**R**V	0
Consensus leader	Blp^&^ pncT	M.TKM.QF.MDT.ML…EGG M.KIDYI.LNEVELETISGG		
ORF67	ComC/BlpC	MKNTVKLEQFKEVTETELQEIRGG	*E*W**R**IP*E*LIRNLIFP**KRK^#^**	+2

* *Some similarity with bacteriocins from S. pyogenes and S. bovis (bovicin-255 variant)*.

§ *https://web.expasy.org/cgi-bin/protparam/protparam*.

& *Not including ORF28 and 45 with a truncated sequence*.

$ *Corresponding genes included in PCR-typing scheme*.

# *CSP, Competence-stimulating peptide. The annotation and consensus is based on S. pneumoniae and Miller et al. (2016). I wrote the amino acids RK in bold and the amino acids DE in bold/italics*.

**Figure 2:**
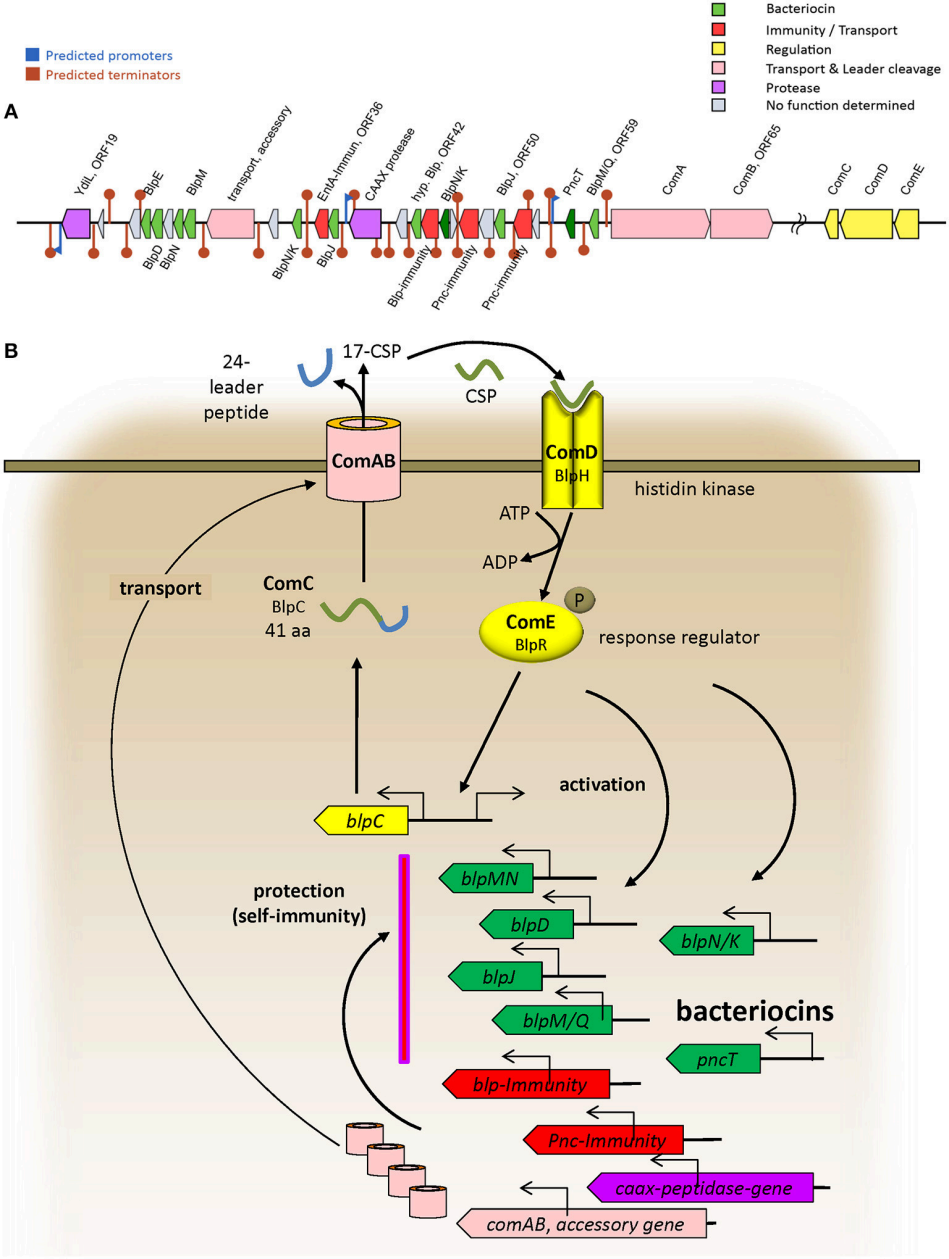
**(A)** Architecture of bacteriocin related operons in the probiotic strain 7746 according to BAGEL4 and improved by manual BLAST search. Only the most representative operons are presented. **(B)** Model of bacteriocin-related protein interactions deduced from Conrads et al. (2014) and Miller et al. (2016).

Taken all results together we annotated the operons of strain 7746 related to bacteriocin-production/transport/immunity as well as to regulation (*comCDE*) and describe a first model of operon architecture and protein/gene interaction (Figure 2; the ComC leader sequence plus mature competence-stimulating-peptide CSP is given in Table 2). For modeling we used a template earlier developed by our group and others (Conrads et al., 2014).

## Discussion

We have developed an isolation procedure for the *Streptococcus oralis* subspecies *dentisani*, abbreviated as *S. dentisani*. Performed in three steps the approach is simple and straight forward. The use of MALDI-TOF may be limiting as not standard in every microbiological laboratory. The eight *S. dentisani* strains isolated during this study showed genetic heterogeneity and diversification, possibly attributed to horizontal gene transfer and/or homologous recombination occurring frequently *within* the oralis and mitis group of streptococci (Kilian et al., 2008; Jensen et al., 2016; Conrads et al., 2017; Rasmussen et al., 2017) but homologous recombination *between* groups is limited to some conserved sequences (Kilian et al., 2008).

It was shown that, apart from colonization of teeth, important probiotic features of *S. dentisani* 7746 are the re-alkalization potential (López-López et al., 2017; Conrads et al., 2018) and its mainly bacteriocin-related antimicrobial properties allowing growth inhibition of important oral pathogens such as *S. mutans, S. sobrinus, Fusobacterium nucleatum* or *Prevotella intermedia* (López-López et al., 2017). However, our results reveal that these antimicrobial effects need reconsideration. First, the inhibition of *S. mutans* (represented by six different strains in the present study) is restricted to low cell density and/or less optimal growth-conditions. As in an initial caries lesion, the number of *S. mutans* cells can be as high as 10^6^ cells—embedded in a matrix of α-D-glucans (mutan) (Pleszczynska et al., 2015; Henne et al., 2016) further inhibiting diffusion—a sufficient action of 7746 bacteriocins *in situ* is at least questionable. In the experiments subjecting two strains of *S. sobrinus* and a single strain of both, *F. nucleatum* and *P. intermedia*, no inhibition could be detected contradicting the results of the Spanish group (López-López et al., 2017). The declaration of a weak or even failed protective action must still be made very carefully as production and activity of bacteriocins is condition-depending (Nagpal et al., 2012), regulated by environmental factors (inoculation sequence, oxygen, nutrition availability, proteases) (Herrero et al., 2016), and due to adaptation and/or mutation-depending on the strain subculture. The 7746-strain subjected here was directly provided by Alex Mira but could, however, still have passed a microevolution.

Furthermore, representative oral conditions are difficult or even impossible to reproduce or mimic *in vitro*. To cover a broad spectrum of factors, we tested several pH-values for growth and bacteriocin production, several atmospheric conditions, three concentration-temperatures to preserve conformation of bacteriocins, additives such as Tween 80 and four different solid as well as two different broth media (with and without sheep or horse blood), but all of these modifications exhibited more-or-less the same results. As we found a minimum of 11 different bacteriocins in the 7746 genome, mostly acting in pairs (class IIb) and requiring equimolar production of two separate peptides and membrane binding, it remains challenging to keep and/or test the optimal bacteriocin activity. Furthermore, it is likely that not all of the detected bacteriocin genes are in general or under certain conditions functionally expressed.

However, the reliability of our testing methods including the principal antimicrobial activity of a *S. dentisani* 7746 concentrate was demonstrated by the strong inhibition we found against other *S. dentisani* strains. This intra-species inhibition reveals a remarkable risk by the consumption of probiotic *S. dentisani* 7746. But, according to our preliminary results, strain 7746 is less potent to out-compete resident caries-protective strains because of cross-resistance. It might replace non-protective *S. dentisani* strains. An outcompeting among streptococcal strains was described before. For instance, recombinant or natural lactate dehydrogenase deficient mutans-streptococci as well as super bacteriocin-producers were shown to replace cariogenic mutans-strains (Hillman et al., 1987, 2000, 2009). Gruner et al. systematically searched randomized controlled trials comparing the efficacy of probiotics vs. control with regards to *S. mutans*, lactobacilli, periodontal pathogens, and clinical symptoms of caries or periodontitis. After a meta-analysis they concluded that application of probiotics for managing gingivitis or periodontitis is advisable but for managing dental caries it is not (Gruner et al., 2016). However, studies subjecting *S. dentisani* were not included and this probiotic strain might overcome some of the limitations because of the triple action including its tropism for teeth. As we and others (Velsko et al., 2018) could demonstrate, the re-alkalization potential of *S. dentisani* 7746 is distinct but the *S. mutans*-directed bacteriocin activity seems to be weak. Furthermore, and worryingly, adaptation/resistance may easily occur, especially after chains of sub-cultivation. Thus, any bacteriocin-depending probiotic strain, especially if marketed, should be taken from a frozen stock, and regularly checked for persistent activity.

As there are many *S. dentisani* strains with different bacteriocin clusters present in nature it is probable to find an even more potent bacteriocin (and ammonia) producer. Through our genome-wide BAGLE4 analysis we were able to identify nine BlpIIb-like bacteriocins, one PncT-like bacteriocin, all originally described in *Streptococcus pneumoniae* as the donor of many genes recombining/transferring in the mitis-oralis group (Kilian et al., 2008; Miller et al., 2016), as well as one bovicin-like bacteriocin originating from *S. bovis* and/or *S. pyogenes*, respectively. Class IIb bacteriocins are acting cooperatively in pairs (Cuozzo et al., 2000; Dawid et al., 2007; Miller et al., 2016) and develop a secondary structure in contact with the target-bacterial membrane, especially when positively charged (Khalaf et al., 2016) as we found applicable for four of them (Table 2, right column**)**. Some of the strain 7746 class IIb bacteriocins are similar to BlpMN from *S. pneumoniae* and to NlmAB from *S. mutans* (Dawid et al., 2007; Kamiya et al., 2011). Many mitis-oralis group strains, including true *S. dentisani* strains RH70047_11 (Accession No. NCUY01000031.1) and UC5873 (Velsko et al., 2018), possess competence regulatory genes with an excessive polymorphism, extensively summarized in literature (Håvarstein et al., 1997; Whatmore et al., 1999; Kilian et al., 2008; Kjos et al., 2016). It should be mentioned that some of the “*S. dentisani*” strains in the NCBI-database were misidentified and incorrectly named, for instance UMB-0008 (*S. oralis*) and UMB_0832 (*S. mitis*), easily to uncover by a 16S- or housekeeping-gene derived phylogenetic tree analysis (Supplementary Figure 2).

Taken together, *S. oralis* subspecies *dentisani*, and especially the probiotic strain 7746 has a large repertoire of bacteriocins with potential to combat and out-compete rivals of pathogenic nature but also resident *S. dentisani* strains and other bacteria of the resident microbiome. As the antimicrobial activity needs a concert of many different small peptides and as mutations on genome level or adaptation on transcriptional level can easily happen, the commercial strains should be regularly tested and chains of sub-cultivation avoided.

## Ethics Statement

A number of 35 caries-free and healthy volunteers were informed about the study and signed an informed consent that had been approved in accordance with the guidelines of the Ethics Committee of the University Hospital Aachen, Germany and the Declaration of Helsinki.

## Author Contributions

GC conceived the work, was implied in the analyses and interpretation of data and wrote the manuscript together with MA. JW, ML, and MA worked in the designing and performance of the experiments, data acquisition, analyses, and interpretation of the results. JW and ML collected the saliva samples. MA and ML made the bioinformatics analyses. All authors edited and approved the manuscript.

### Conflict of Interest Statement

The authors declare that the research was conducted in the absence of any commercial or financial relationships that could be construed as a potential conflict of interest.

## References

[B1] Alikhan N. F. Petty N. K. Ben Zakour N. L. Beatson S. A. (2011). BLAST Ring Image Generator (BRIG): simple prokaryote genome comparisons. BMC Genomics 12:402. 10.1186/1471-2164-12-402 21824423 PMC3163573

[B2] Badet C. Thebaud N. B. (2008). Ecology of lactobacilli in the oral cavity: a review of literature. Open Microbiol. J. 2, 38–48. 10.2174/1874285800802010038 19088910 PMC2593047

[B3] Conrads G. Barth S. Möckel M. Lenz L. van der Linden M. Henne K. (2017). *Streptococcus tigurinus* is frequent among *gtfR*-negative *Streptococcus oralis* isolates and in the human oral cavity, but highly virulent strains are uncommon. J. Oral Microbiol. 9:1307079. 10.1080/20002297.2017.1307079 28473881 PMC5405715

[B4] Conrads G. Bockwoldt J. A. Kniebs C. Abdelbary M. M. H. (2018). Commentary: Health-Associated Niche Inhabitants as Oral Probiotics: the case of *Streptococcus dentisani*. Front. Microbiol. 9:340. 10.3389/fmicb.2018.00340 29535701 PMC5835103

[B5] Conrads G. de Soet J. J. Song L. Henne K. Sztajer H. Wagner-Döbler I.. (2014). Comparing the cariogenic species *Streptococcus sobrinus* and *S. mutans* on whole genome level. J. Oral Microbiol. 6:26189. 10.3402/jom.v6.26189 25475081 PMC4256546

[B6] Cuozzo S. A. Sesma F. Palacios J. M. de Ruíz Holgado A. P. Raya R. R. (2000). Identification and nucleotide sequence of genes involved in the synthesis of lactocin 705, a two-peptide bacteriocin from *Lactobacillus casei* CRL 705. FEMS Microbiol. Lett. 185, 157–161. 10.1111/j.1574-6968.2000.tb09055.x 10754241

[B7] Darling A. C. Mau B. Blattner F. R. Perna N. T. (2004). Mauve: multiple alignment of conserved genomic sequence with rearrangements. Genome Res. 14, 1394–1403. 10.1101/gr.2289704 15231754 PMC442156

[B8] Darling A. E. Mau B. Perna N. T. (2010). progressiveMauve: multiple genome alignment with gene gain, loss and rearrangement. PLoS ONE 5:e11147. 10.1371/journal.pone.0011147 20593022 PMC2892488

[B9] Dawid S. Roche A. M. Weiser J. N. (2007). The blp bacteriocins of *Streptococcus pneumoniae* mediate intraspecies competition both *in vitro* and *in vivo*. Infect. Immun. 75, 443–451. 10.1128/IAI.01775-05 17074857 PMC1828380

[B10] de Jong A. van Hijum S. A. Bijlsma J. J. Kok J. Kuipers O. P. (2006). BAGEL: a web-based bacteriocin genome mining tool. Nucleic Acids Res. 34:W273–W279. 10.1093/nar/gkl237 16845009 PMC1538908

[B11] Di Pierro F. Zanvit A. Nobili P. Risso P. Fornaini C. (2015). Cariogram outcome after 90 days of oral treatment with *Streptococcus salivarius* M18 in children at high risk for dental caries: results of a randomized, controlled study. Clin. Cosmet. Investig. Dent. 7, 107–113. 10.2147/CCIDE.S93066 26491371 PMC4598214

[B12] Global Burden of Disease Study C. (2015). Global, regional, and national incidence, prevalence, and years lived with disability for 301 acute and chronic diseases and injuries in 188 countries, 1990-2013: a systematic analysis for the Global Burden of Disease Study 2013. Lancet 386, 743–800. 10.1016/S0140-6736(15)60692-4 26063472 PMC4561509

[B13] Gruner D. Paris S. Schwendicke F. (2016). Probiotics for managing caries and periodontitis: systematic review and meta-analysis. J. Dent. 48, 16–25. 10.1016/j.jdent.2016.03.002 26965080

[B14] Håvarstein L. S. Hakenbeck R. Gaustad P. (1997). Natural competence in the genus *Streptococcus*: evidence that streptococci can change pherotype by interspecies recombinational exchanges. J. Bacteriol. 179, 6589–6594. 9352904 10.1128/jb.179.21.6589-6594.1997 PMC179583

[B15] Henne K. Gunesch A. P. Walther C. Meyer-Lueckel H. Conrads G. Esteves-Oliveira M. (2016). Analysis of bacterial activity in sound and cariogenic biofilm: a pilot *in vivo* study. Caries Res. 50, 480–488. 10.1159/000448485 27595541

[B16] Herrero E. R. Slomka V. Bernaerts K. Boon N. Hernandez-Sanabria E. Passoni B. B.. (2016). Antimicrobial effects of commensal oral species are regulated by environmental factors. J. Dent. 47, 23–33. 10.1016/j.jdent.2016.02.007 26875613

[B17] Hillman J. D. Brooks T. A. Michalek S. M. Harmon C. C. Snoep J. L. van Der Weijden C. C. (2000). Construction and characterization of an effector strain of *Streptococcus mutans* for replacement therapy of dental caries. Infect. Immun. 68, 543–549. 10.1128/IAI.68.2.543-549.2000 10639415 PMC97174

[B18] Hillman J. D. Dzuback A. L. Andrews S. W. (1987). Colonization of the human oral cavity by a *Streptococcus mutans* mutant producing increased bacteriocin. J. Dent. Res. 66, 1092–1094. 10.1177/00220345870660060101 3476580

[B19] Hillman J. D. McDonell E. Cramm T. Hillman C. H. Zahradnik R. T. (2009). A spontaneous lactate dehydrogenase deficient mutant of *Streptococcus rattus* for use as a probiotic in the prevention of dental caries. J. Appl. Microbiol. 107, 1551–1558. 10.1111/j.1365-2672.2009.04333.x 19426263

[B20] Horz H. P. Meinelt A. Houben B. Conrads G. (2007). Distribution and persistence of probiotic *Streptococcus salivarius* K12 in the human oral cavity as determined by real-time quantitative polymerase chain reaction. Oral Microbiol. Immunol. 22, 126–130. 10.1111/j.1399-302X.2007.00334.x 17311636

[B21] Huang X. Browngardt C. M. Jiang M. Ahn S. J. Burne R. A. Nascimento M. M. (2018). Diversity in antagonistic interactions between commensal oral streptococci and *Streptococcus mutans*. Caries Res. 52, 88–101. 10.1159/000479091 29258070 PMC5828942

[B22] Hyink O. Wescombe P. A. Upton M. Ragland N. Burton J. P. Tagg J. R. (2007). Salivaricin A2 and the novel lantibiotic salivaricin B are encoded at adjacent loci on a 190-kilobase transmissible megaplasmid in the oral probiotic strain *Streptococcus salivarius* K12. Appl. Environ. Microbiol. 73, 1107–1113. 10.1128/AEM.02265-06 17194838 PMC1828679

[B23] Jensen A. Scholz C. F. Kilian M. (2016). Re-evaluation of the taxonomy of the Mitis group of the genus *Streptococcus* based on whole genome phylogenetic analyses, and proposed reclassification of *Streptococcus dentisani* as *Streptococcus oralis* subsp. *dentisani* comb. nov., *Streptococcus tigurinus* as *Streptococcus oralis* subsp. tigurinus comb. nov., and *Streptococcus oligofermentans* as a later synonym of *Streptococcus cristatus*. Int. J. Syst. Evol. Microbiol. 66, 4803–4820. 10.1099/ijsem.0.001433 27534397

[B24] Jindal G. Pandey R. K. Agarwal J. Singh M. (2011). A comparative evaluation of probiotics on salivary mutans streptococci counts in Indian children. Eur. Arch. Paediatr. Dent. 12, 211–215. 10.1007/BF03262809 21806906

[B25] Kamiya R. U. Taiete T. Gonçalves R. B. (2011). Mutacins of *Streptococcus mutans*. Braz. J. Microbiol. 42, 1248–1258. 10.1590/S1517-83822011000400001 24031748 PMC3768731

[B26] Khalaf H. Nakka S. S. Sandén C. Svärd A. Hultenby K. Scherbak N.. (2016). Antibacterial effects of *Lactobacillus* and bacteriocin PLNC8 alphabeta on the periodontal pathogen *Porphyromonas gingivalis*. BMC Microbiol. 16:188. 10.1186/s12866-016-0810-8 27538539 PMC4990846

[B27] Kilian M. Poulsen K. Blomqvist T. Håvarstein L. S. Bek-Thomsen M. Tettelin H.. (2008). Evolution of *Streptococcus pneumoniae* and its close commensal relatives. PLoS ONE 3:e2683. 10.1371/journal.pone.0002683 18628950 PMC2444020

[B28] Kjos M. Miller E. Slager J. Lake F. B. Gericke O. Roberts I. S.. (2016). Expression of *Streptococcus pneumoniae* bacteriocins is induced by antibiotics via regulatory interplay with the competence system. PLoS Pathog. 12:e1005422. 10.1371/journal.ppat.1005422 26840404 PMC4739728

[B29] López-López A. Camelo-Castillo A. Ferrer M. D. Simon-Soro A. Mira A. (2017). Health-Associated niche inhabitants as oral probiotics: the case of *Streptococcus dentisani*. Front. Microbiol. 8:379. 10.3389/fmicb.2017.00379 28344574 PMC5344910

[B30] Mantzourani M. Gilbert S. C. Sulong H. N. Sheehy E. C. Tank S. Fenlon M.. (2009). The isolation of bifidobacteria from occlusal carious lesions in children and adults. Caries Res. 43, 308–313. 10.1159/000222659 19494490

[B31] Miller E. L. Abrudan M. I. Roberts I. S. Rozen D. E. (2016). Diverse ecological strategies are encoded by *Streptococcus pneumoniae* bacteriocin-like peptides. Genome Biol. Evol. 8, 1072–1090. 10.1093/gbe/evw055 26983823 PMC4860687

[B32] Nagpal R. Kumar A. Kumar M. Behare P. V. Jain S. Yadav H. (2012). Probiotics, their health benefits and applications for developing healthier foods: a review. FEMS Microbiol. Lett. 334, 1–15. 10.1111/j.1574-6968.2012.02593.x 22568660

[B33] Pham L. C. van Spanning R. J. Röling W. F. Prosperi A. C. Terefework Z. Ten Cate J. M.. (2009). Effects of probiotic *Lactobacillus salivarius* W24 on the compositional stability of oral microbial communities. Arch. Oral Biol. 54, 132–137. 10.1016/j.archoralbio.2008.09.007 18976742

[B34] Pleszczynska M. Wiater A. Janczarek M. Szczodrak J. (2015). (1–>3)-α-D-Glucan hydrolases in dental biofilm prevention and control: a review. Int. J. Biol. Macromol. 79, 761–778. 10.1016/j.ijbiomac.2015.05.052 26047901

[B35] Rasmussen L. H. Højholt K. Dargis R. Christensen J. J. Skovgaard O. Justesen U. S. (2017). *In silico* assessment of virulence factors in strains of *Streptococcus oralis* and *Streptococcus mitis* isolated from patients with infective endocarditis. J. Med. Microbiol. 66, 1316–1323d 10.1099/jmm.0.000573 28874232

[B36] Tagg J. R. Bannister L. V. (1979). “Fingerprinting” beta-haemolytic streptococci by their production of and sensitivity to bacteriocine-like inhibitors. J. Med. Microbiol. 12, 397–411. 10.1099/00222615-12-4-397 41951

[B37] Thurnheer T. Belibasakis G. N. (2018). *Streptococcus oralis* maintains homeostasis in oral biofilms by antagonizing the cariogenic pathogen *Streptococcus mutans*. Mol. Oral Microbiol. 33, 234–239. 10.1111/omi.12216 29327482

[B38] Untergasser A. Nijveen H. Rao X. Bisseling T. Geurts R. Leunissen J. A. (2007). Primer3Plus, an enhanced web interface to Primer3. Nucleic Acids Res. 35, W71–W74. 10.1093/nar/gkm306 17485472 PMC1933133

[B39] van Heel A. J. de Jong A. Song C. Viel J. H. Kok J. Kuipers O. P. (2018). BAGEL4: a user-friendly web server to thoroughly mine RiPPs and bacteriocins. Nucleic Acids Res. 46, W278–W281. 10.1093/nar/gky383 29788290 PMC6030817

[B40] Velsko I. M. Chakraborty B. Nascimento M. M. Burne R. A. Richards V. P. (2018). Species designations belie phenotypic and genotypic heterogeneity in oral streptococci. mSystems 3, e00158–18. 10.1128/mSystems.00158-18 30574560 PMC6299155

[B41] Wescombe P. A. Hale J. D. Heng N. C. Tagg J. R. (2012). Developing oral probiotics from *Streptococcus salivarius*. Future Microbiol. 7, 1355–1371. 10.2217/fmb.12.113 23231486

[B42] Whatmore A. M. Barcus V. A. Dowson C. G. (1999). Genetic diversity of the streptococcal competence (com) gene locus. J. Bacteriol. 181, 3144–3154 10322016 10.1128/jb.181.10.3144-3154.1999 PMC93770

[B43] Zahradnik R. T. Magnusson I. Walker C. McDonell E. Hillman C. H. Hillman J. D. (2009). Preliminary assessment of safety and effectiveness in humans of ProBiora3, a probiotic mouthwash. J. Appl. Microbiol. 107, 682–690. 10.1111/j.1365-2672.2009.04243.x 19486429

