# Supplementary material for: Isolation and Bacteriocin-Related Typing of Streptococcus dentisani
Source: Front Cell Infect Microbiol. 2019 Apr 16;9:110. doi: 10.3389/fcimb.2019.00110 (PMC6476965; doi:10.3389/fcimb.2019.00110)
Supplement: Supplementary file 1 [file Table_1.DOCX]

**SUPPLEMENTARY TABLE 1** Strains subjected in this study and their origin and function (test strain and/or producer).

| **Genus** | **Species** | **Strain number** | **Material**  **(all human)** | **Origin**  **(depositor name / place)** | **Function** |
| --- | --- | --- | --- | --- | --- |
| *Aggregatibacter* | *actinomycetem-comitans* | ATCC 33384 | Abscess | London | test |
| *Capnocytophaga* | *ochracea* | ATCC 27872 | Oral cavity | Cato & Holdemann | test |
| *Fusobacterium* | *nucleatum* | ATCC 25586 | Cervico-facial lesion | Prevot | test |
| *Lactobacillus* | *casei* | DSM 20020 | Saliva | Rogosa | test |
| *Porphyromonas* | *gingivalis* | ATCC 33277 | Gingival sulcus | Owen | test |
| *Prevotella* | *intermedia* | ATCC 25611 | Empyema | Holdemann & Moore | test |
| *Streptococcus* | *anginosus* | OMI 327 | Feaces | Aachen | test |
| *Streptococcus* | *constellatus* | AC/1243/1 | Abscess | Aachen | test |
| *Streptococcus* | *dentisani* | 7746 | Plaque | Mira / Spain | producer/ test |
| *Streptococcus* | *dentisani* | 7747^T^  OMI 276-283* | Plaque | Mira / Spain | producer/ test |
| *Streptococcus* | *dentisani* | OMI 105  SN54788 | [Endocarditis](https://www.dict.cc/englisch-deutsch/endocarditis.html) | NRZ, Aachen | producer/  test |
| *Streptococcus* | *dentisani* | OMI 116  SN39325 | [Endocarditis](https://www.dict.cc/englisch-deutsch/endocarditis.html) | NRZ, Aachen | producer/  test |
| *Streptococcus* | *dentisani* | OMI 166  SN58364 | [Endocarditis](https://www.dict.cc/englisch-deutsch/endocarditis.html) | NRZ, Aachen | producer/  test |
| *Streptococcus* | *dentisani* | OMI 168  SN54787 | [Endocarditis](https://www.dict.cc/englisch-deutsch/endocarditis.html) | Aachen | producer/  test |
| *Streptococcus* | *dentisani* | OMI 214 | Dental biofilm | Aachen | producer/  test |
| *Streptococcus* | *dentisani* | OMI 215 | Dental biofilm | Aachen | producer/  test |
| *Streptococcus* | *dentisani* | OMI 284 | Saliva | Aachen | producer/ test |
| *Streptococcus* | *dentisani* | OMI 285 | Saliva | Aachen | producer/ test |
| *Streptococcus* | *dentisani* | OMI 287 | Saliva | Aachen | producer/  test |
| *Streptococcus* | *dentisani* | OMI 288 | Saliva | Aachen | producer/  test |
| *Streptococcus* | *dentisani* | OMI 290  a and b | Saliva | Aachen | producer/ test |
| *Streptococcus* | *dentisani* | OMI 291 | Saliva | Aachen | producer/ test |
| *Streptococcus* | *dentisani* | OMI 310 | Saliva | Aachen | test |
| *Streptococcus* | *gallolyticus* | OMI 326 | Feaces | Aachen | test |
| *Streptococcus* | *infantis* | OMI 289 | Saliva | Aachen | test |
| *Streptococcus* | *mitis/oralis/*  *peroris/*  *pneumoniae* ^a.)^ | OMI 337 | Saliva | Aachen | test |
| *Streptococcus* | *mutans* | AC 4446 | [Endocarditis](https://www.dict.cc/englisch-deutsch/endocarditis.html) | Aachen | test |
| *Streptococcus* | *mutans* | ATCC 25175^T^ | Carious dentine | London | test |
| *Streptococcus* | *mutans* | KK 5/21 | Caries (child) | Kneist / Jena | test |
| *Streptococcus* | *mutans* | KK 5/23 | Caries (child) | Kneist / Jena | test |
| *Streptococcus* | *mutans* | R658 | Caries (child) | Kneist / Jena | test |
| *Streptococcus* | *mutans* | UA159 | Caries (child) | Caufield | test |
| *Streptococcus* | *oralis* | OMI 334 | Saliva | Aachen | test |
| *Streptococcus* | *parasanguinis* | OMI 329 | Saliva | Aachen | test |
| *Streptococcus* | *parasanguinis* | OMI 335 | Saliva | Aachen | test |
| *Streptococcus* | *peroris/mitis* ^a.)^ | OMI 333 | Saliva | Aachen | test |
| *Streptococcus* | *pneumoniae* | OMI 330 | Saliva | Aachen | test |
| *Streptococcus* | *pseudopneumoniae/oralis/*  *pneumoniae/*  *peroris* ^a.)^ | OMI 336 | Saliva | Aachen | test |
| *Streptococcus* | *salivarius* | OMI 315 | Saliva | Aachen | test |
| *Streptococcus* | *salivarius/*  *peroris/*  *pneumoniae/*  *oralis)* ^a.)^ | OMI 331 | Saliva | Aachen | test |
| *Streptococcus* | *sanguinis* | OMI 332 | Saliva | Aachen | test |
| *Streptococcus* | *sobrinus* | AC 163 | Biofilm | Aachen | test |
| *Streptococcus* | *sobrinus* | ATCC 33478 | Dental biofilm | Kocur | test |
| *Streptococcus* | *vestibularis* | OMI 238 | Saliva | Aachen | test |

a) The first species mentioned is the priority result in MALDI-TOF MS.

* bacteriocin resistant/adapted variants.
