# Supplementary figures and images for: Isolation and Bacteriocin-Related Typing of Streptococcus dentisani
Source: Front Cell Infect Microbiol. 2019 Apr 16;9:110. doi: 10.3389/fcimb.2019.00110 (PMC6476965; doi:10.3389/fcimb.2019.00110)

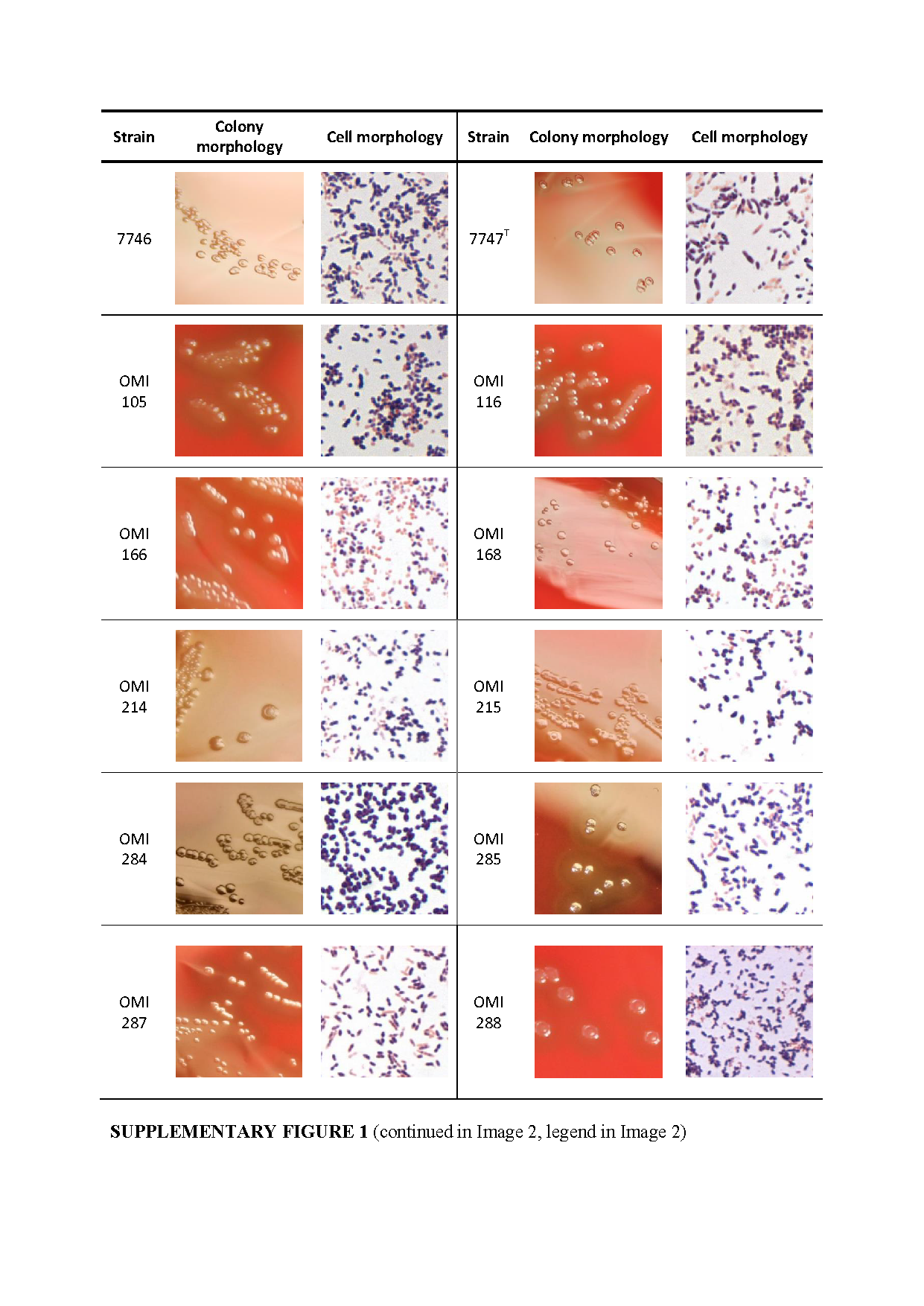

Supplement: Supplementary file 4 [file Image_1.tif]

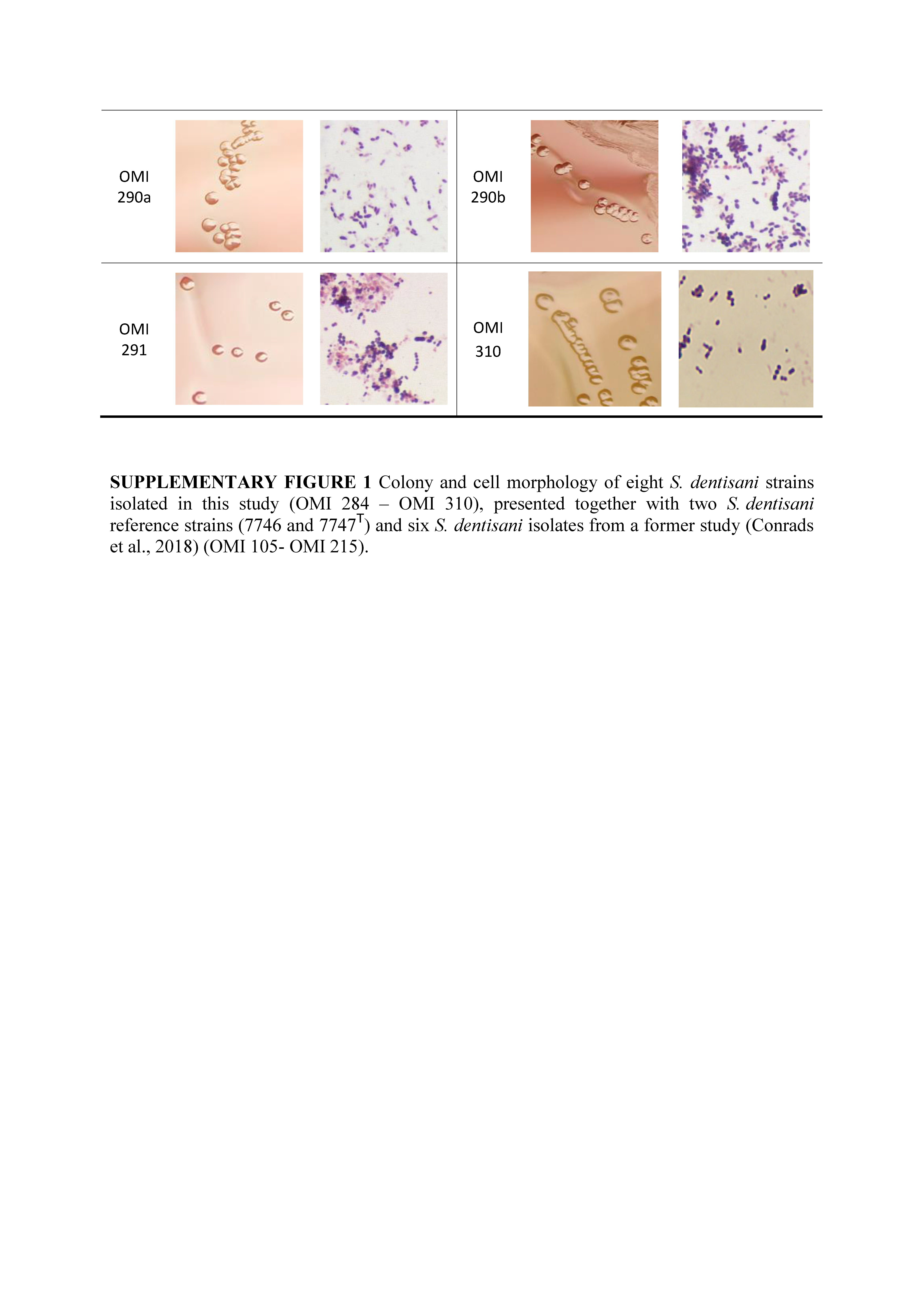

Supplement: Supplementary file 5 [file Image_2.TIFF]

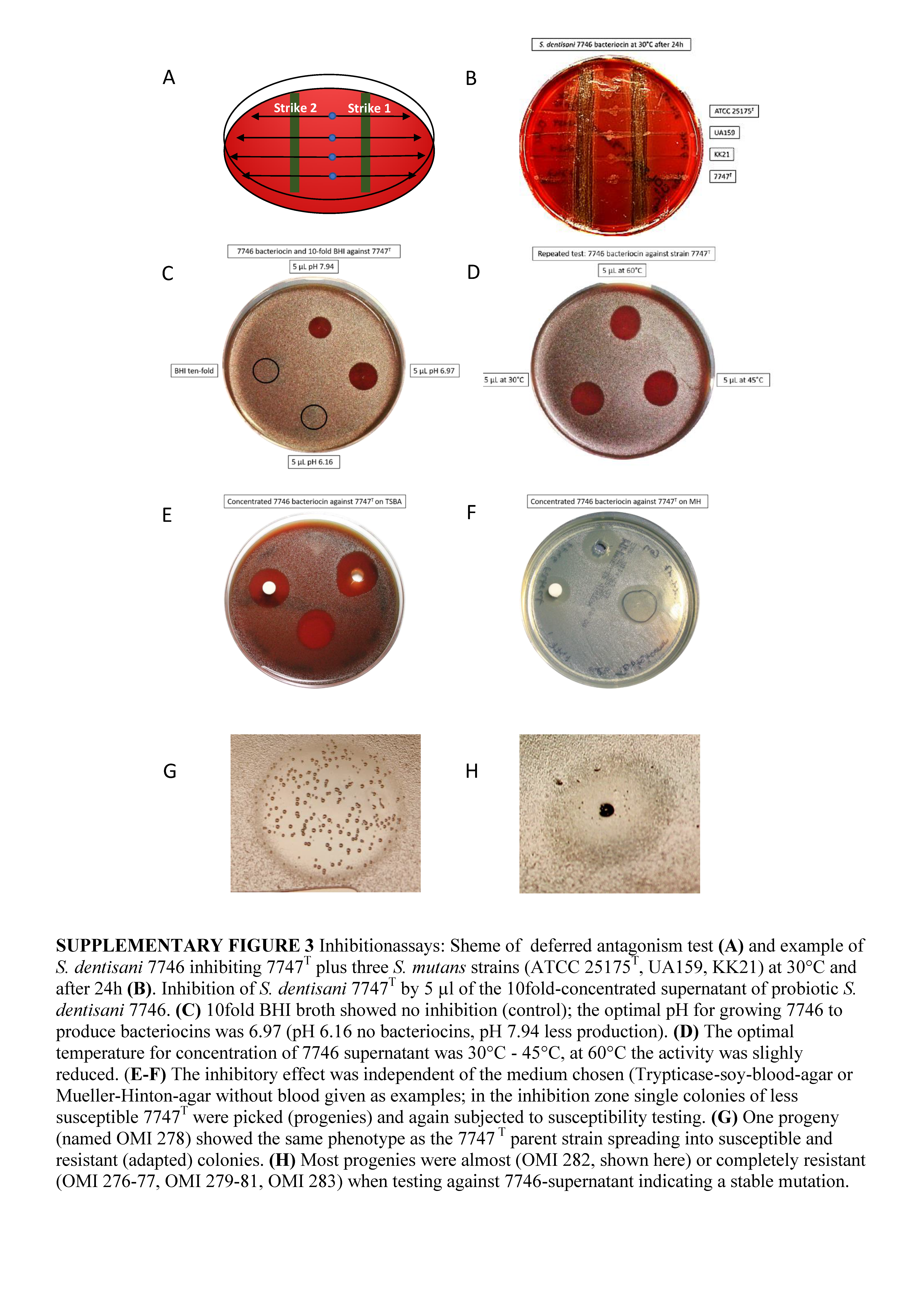

Supplement: Supplementary file 7 [file Image_4.TIFF]
